# The Pyramiding of Three Key Root Traits Aid Breeding of Flood-Tolerant Rice

**DOI:** 10.3390/plants11152033

**Published:** 2022-08-04

**Authors:** Chen Lin, Tongtong Zhu, Lucas León Peralta Ogorek, Youping Wang, Margret Sauter, Ole Pedersen

**Affiliations:** 1 Key Laboratory of Plant Functional Genomics of the Ministry of Education, Yangzhou University, Yangzhou 225009, China; 2 Freshwater Biological Laboratory, Department of Biology, University of Copenhagen, 2100 Copenhagen, Denmark; 3 Jiangsu Key Laboratory of Crop Genomics and Molecular Breeding, Yangzhou University, Yangzhou 225009, China; 4 Plant Developmental Biology and Plant Physiology, University of Kiel, Am Botanischen Garten 5, 24118 Kiel, Germany

**Keywords:** adventitious root, aerenchyma formation, flooding, barrier to radial oxygen loss, rice, ROL

## Abstract

Flooding is constantly threatening the growth and yield of crops worldwide. When flooding kicks in, the soil becomes water-saturated and, therefore, the roots are the first organs to be exposed to excess water. Soon after flooding, the soil turns anoxic and the roots can no longer obtain molecular oxygen for respiration from the rhizosphere, rendering the roots dysfunctional. Rice, however, is a semi-aquatic plant and therefore relatively tolerant to flooding due to adaptive traits developed during evolution. In the present review, we have identified three key root traits, viz. cortical aerenchyma formation, a barrier to radial oxygen loss and adventitious root growth. The understanding of the physiological function, the molecular mechanisms, and the genetic regulation of these three traits has grown substantially and therefore forms the backbone of this review. Our synthesis of the recent literature shows each of the three key root traits contributes to flood tolerance in rice. One trait, however, is generally insufficient to enhance plant tolerance to flooding. Consequently, we suggest comprehensive use of all three adaptive traits in a pyramiding approach in order to improve tolerance to flooding in our major crops, in general, and in rice, in particular.

## 1. Introduction

The food demand is increasing annually owing to the world’s growing population [1]. In the last decades, the growth and yield of crops have been simultaneously threatened by various natural disasters, and among them are frequent flooding events [2]. The cultivation and breeding of flooding-tolerant plant varieties are thus becoming the prime task to alleviate the food crisis caused by soil flooding or even complete submergence. Wetland plants oftentimes exhibit higher waterlogging tolerance due to their evolutionary ability to thrive in such habitats, and various adaptive traits have been described in these plants, conferring tolerance to flash floods or prolonged flood events [3].

**Figure 1:**
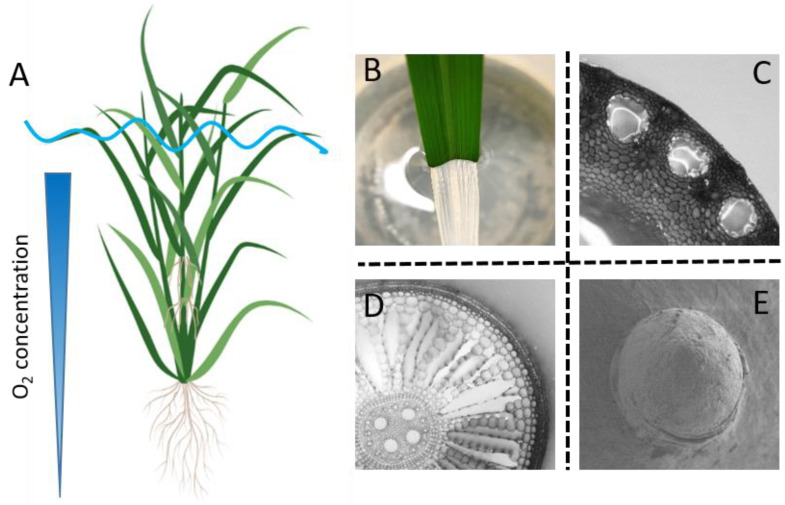
Rice adaptive traits during waterlogging. Rice develops a range of adaptive traits upon soil flooding including stem elongation (**A**), gas film retention by the superhydrophobic leaves (**B**), aerenchyma formation in the internodes of the stem (**C**) and in the cortex of the roots (**D**), and floating adventitious roots (**A**,**E**). Panel (**A**) is created with BioRender.com, and photos and cross-sections are all original contributions by the authors.

Rice is a semi-aquatic plant that survives soil flooding or even partial submergence. It possesses a range of adaptive traits to cope with floods, such as stem elongation [4], gas film retention by superhydrophobic leaves [5], aerenchyma formation in internodes [6] as well as in roots [7], and adventitious root growth [8,9] (Figure 1). Adaptive root traits are receiving considerable interest due to their high plasticity [10] and due to the fact that roots are more prone to soil flooding as compared to the shoot. In water-saturated (waterlogged) soils, O_2_ supply from the air to the soil is greatly restricted due to high resistance to molecular diffusion in water compared to in air. Moreover, the soil roots and microorganisms compete for the remaining O_2_, making the situation even more severe [9]. Lack of O_2_ inhibits root respiration, causing severe energy crisis and finally resulting in tissue death [11]. Consequently, the introgression of key root traits in the soil roots and the formation of a new root system are seen as two major strategies in rice to cope with soil flooding and partial submergence.

Understanding the function of these adaptive traits is helpful to uncover the underlying mechanisms of plant adaptation to flooding, thus enabling the valuable quantitative trait loci (QTLs) or genes associated with these traits to be introgressed into flooding-susceptible genotypes through traditional hybridization or genetic engineering [12]. In this review, we summarize the progress in recent research of three important adaptive root traits expressed in rice and some other crops as a response to flooding: (i) cortical aerenchyma formation, (ii) the root barrier to radial oxygen loss, and (iii) aquatic adventitious root formation. The aim of this review is, therefore, to analyze recent research enabling breeding of climate-resilient rice based on pyramiding these three key root traits to optimize adaptation to flooding.

## 2. Aerenchyma Formation Facilitates Intra-Tissue O_2_ Diffusion in Flooded Soil Roots

**Figure 2:**
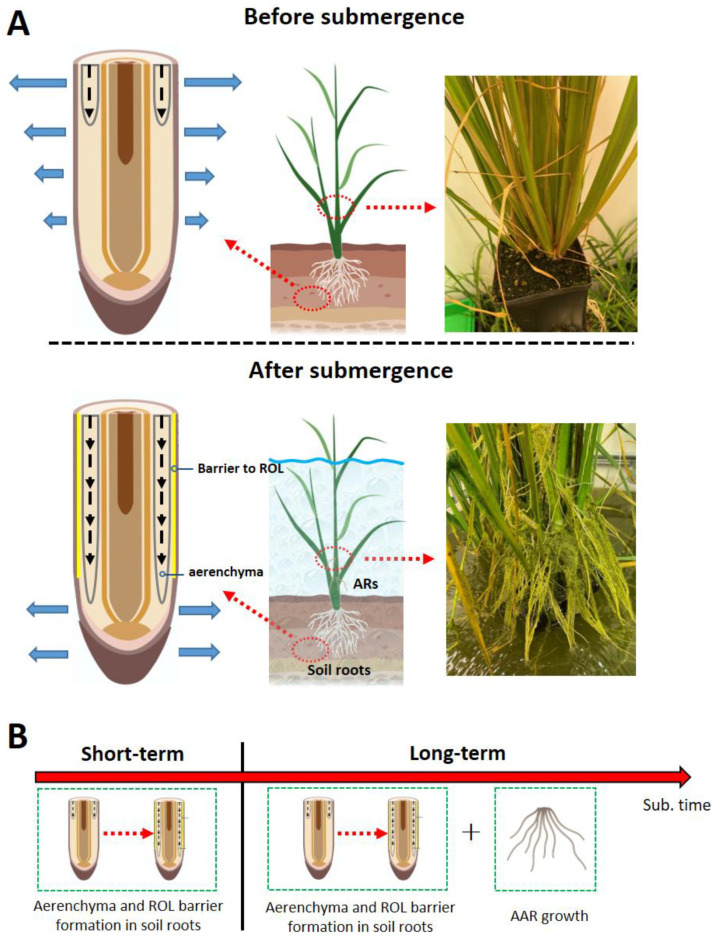
Three adaptive root traits conferring tolerance to soil flooding in rice. (**A**) The inducible aerenchyma is developed inside the soil roots to enhance O_2_ diffusion from the shoot to the root. A barrier to radial oxygen loss (ROL) is formed at the outer part of the soil roots to restrict loss of molecular O_2_ to the anoxic rhizosphere. Furthermore, the shoot-borne aquatic adventitious roots (AARs) are formed aboveground. (**B**) Roots adaptive traits occur as the flooding progresses. Several components are created with BioRender.com.

Aerenchyma consist of gas-filled plant tissues, and the formation of cortical aerenchyma at the base of rice roots, for example, results from the lysis of cortex cells [13]. Root aerenchyma can either be constitutively formed or further induced as a response to the soil environment [14]. Aerenchyma is a key root trait enabling plants to survive flooded soils, because the gas-filled tissues provide a low-resistance pathway for O_2_ diffusion inside the roots, thus supplying molecular O_2_ to the growing root tip [15] (Figure 2A). Moreover, the programmed death of cortical cells reduces the root respiratory consumption of carbohydrates, thereby conserving organic carbon and nutrients, i.e., a longer root can be produced for the same investment in carbon and nutrients if a large proportion of the cortex is gas-filled [13,16]. In recent years, the molecular mechanism of aerenchyma formation in rice has gradually been uncovered. In rice, root cortical aerenchyma is constitutively formed [17], but great differences in constitutive aerenchyma have been found among genotypes and, interestingly, these differences have not been strongly correlated to their flooding tolerance. Non-wetland plants such as maize (*Zea mays*) and wheat (*Triticum aestivum*) barely form any constitutive aerenchyma and their roots easily suffer from tissue anoxia at the onset of waterlogging [17,18]. It is thus conceivable that constitutive aerenchyma formation in rice is closely associated with tolerance to waterlogging. Genetically, formation of constitutive aerenchyma in rice is regulated by auxin signaling. Auxin/indole-3-acetic acid protein IAA13 and the auxin response factor ARF19 together contribute to the development of constitutive aerenchyma by directly targeting the downstream transcription factor lateral organ boundaries domain (LBD)-containing protein 1–8 (LBD1–8), which is also required for constitutive aerenchyma formation [19].

In stark contrast, the inducible aerenchyma follows an ethylene-ROS regulatory manner upon soil flooding [7]. At the onset of flooding, the elevated ethylene in plant roots is a combination of slow gas diffusion (causing the constitutive production of ethylene to accumulate in the tissues) and upregulation of the ethylene biosynthesis genes, ACC synthase (ACS) and ACC oxidase (ACO) [20,21]. The expression of *ACS1* and *ACO5* is strongly induced in stagnant, deoxygenated hydroponics designed to mimic soil flooding [20], and the inhibition of ethylene biosynthesis and signaling significantly repressed the formation of inducible aerenchyma [20]. At later stages, ROS accumulation is also required for the formation of inducible aerenchyma [7]. Respiratory Burst Oxidase Homolog (RBOH) is the homolog of NADPH oxidase, playing a pivotal role in ROS generation in the apoplast; the accumulated ROS subsequently enters the cytosol. Expression of one of the RBOH isoforms (RBOHH) is induced in stagnant, deoxygenated conditions, and knockout of RBOHH significantly reduced the ROS production and aerenchyma formation [7]. Moreover, Ca^2+^-dependent signaling is needed for the induction of inducible aerenchyma [7]. Two calcium-dependent protein kinases (CPK/CDPK) CDPK5 and CDPK13 are crucial for the phosphorylation of RBOHH [7]. In addition, application of the RBOH inhibitor, diphenyleneiodonium (DPI), inhibited the formation of inducible aerenchyma as well. In conclusion, the formation of inducible aerenchyma is under strong control of ethylene, ROS, and Ca^2+^-dependent signaling.

## 3. Some Roots Develop a Barrier to Radial Oxygen Loss to Facilitate Internal Aeration

Due to the steep concentration gradient, oxygen entrapped in the root aerenchyma tends to diffuse radially to the surrounding anoxic soil rather than longitudinally to the root tip. In rice and other wetland plants, radial oxygen loss (ROL) from roots can be greatly restricted by the formation of a diffusive barrier at the exodermis (epidermal/hypodermal cell layers) when exposed to conditions of soil flooding or partial submergence [22,23]. The root barrier to ROL not only improves tissue oxygen status by restricting diffusive loss of oxygen, but also prevents soil phytotoxins from entering the root [24]. The ROL barrier is also capable of restricting the diffusion of gases other than O_2_, such as H_2_ or water vapour [25]. The root ROL barrier is presumably composed of enhanced cell wall depositions of suberin and/or lignin at the exodermis [26]. The location of the ROL barrier in the exodermis is supported by measurement using oxygen microsensors, indicating steep concentration gradients in the outer cell layers (epidermis, exodermis/hypodermis and sclerenchyma) [27]. Not only the exact components of the ROL barrier are still being debated, but it is also not yet clear which of the two known components (suberin and/or lignin) is of greater significance to reduce ROL in rice [27]. However, in four Amazonian tree species [28] and *Hordeum marinum* [29], it was found that suberization of the exodermis, more than lignification, was correlated with a reduction in ROL.

The formation of an ROL barrier is influenced by various environmental factors. Soil flooding leads to oxygen deprivation, accumulation of the phytohormone ethylene and also CO_2_, but none of these molecules act as an environmental signal(s) for ROL barrier formation [30]. In contrast, some soil phytotoxins such as Fe^2+^ [31], sulfides [32], and low molecular carboxylic acids [33] produced by anaerobic bacteria all induce a ROL barrier in rice. Interestingly, the molecular regulation of the ROL barrier formation in plants is still under investigation. By means of laser microdissection, the specific tissue where the ROL barrier is formed can be collected and used for transcriptome analysis [26]. Several genes involved in suberin biosynthesis, including *CYTOCHROME P450* (*OsCYP86B3*) and *ABC TRANSPORTER* (*OsABCG5*), are strongly induced under soil flooding [26], suggesting that these are likely important molecular regulators required for ROL barrier formation.

In root tissues of the *OsCYP86B3* rice mutant, the amount of C24 to C30 w/OH fatty acid was much lower compared to the wildtype, whereas the suberin lamellae were still detectable with histochemical staining [34]. In addition, oxygen leakage was observed at the root tip rather than at the basal part of the root, indicating that the lack of *OsCYP86B3* did not influence the formation of the ROL barrier, and the knockout of *OsCYP86B3* did also not alter root physiology. In contrast, *OsABCG5* mutant plants also show a much lower C24 to C30 w/OH fatty acid, but unlike the *OsCYP86B3* rice mutant, suberin lamellae was not histochemically detected in the *OsACCG5* mutant and importantly, the apoplastic barrier was impaired [35]. To date, the ROL barrier in *OsABCG5* mutant plants has not been systematically evaluated. In addition, the *OsACCG5* mutant showed shorter roots as compared to the wildtype, indicating additional roles of *OsACCG5* in root development.

Several transcription factors including WRKY, NAC, and MYB were upregulated under stagnant, deoxygenated conditions (simulating soil flooding), indicating that these transcription factors might be directly or indirectly involved in the ROL barrier formation [26]. Owning to the lack of relevant rice mutants, the functional analysis of these genes as related to the ROL barrier formation remains to be investigated. Recently, it was demonstrated that Abscisic Acid (ABA) signaling is required for ROL barrier formation, whereas the application of the ABA biosynthesis inhibitor FLU prevented the formation of ROL barrier. *OsABA1* shows an impaired ROL barrier, but when complemented with ABA, it is perfectly able to form a ROL barrier under stagnant, deoxygenated conditions [36]. We therefore propose to investigate other ABA-related genes in order to further unravel the genes involved in the ROL barrier formation.

## 4. Aquatic Adventitious Roots Confer Tolerance to Partial or Complete Submergence

Primordia of adventitious roots (AR) are present in the nodes of the aboveground parts of the stem in rice but do not emerge unless triggered by environmental factors such as soil flooding or partial submergence [8,37]. Some emerging roots penetrate into the soil, whereas others float in the water, and these are therefore referred to as aquatic ARs (AAR) [38]. Growth of ARs is coordinated with programmed cell death above the root primordia and induced by the gaseous phytohormone ethylene [39,40]. The new AR system alleviates the functional loss of soil roots, thus helping plants to endure flooding until the water recedes. AARs of deepwater rice have been shown to actively take up nutrients from the floodwater [38,41], and unlike the main roots exposed to anoxic soil, AAR can still obtain oxygen dissolved in the floodwater [42]. Interestingly, in several wild wetland plants, functional chloroplasts develop in AAR, indicating that oxygen and carbohydrates are produced by these roots [42,43]. During long-term and periodic flooding, the AARs are therefore of great importance to ensure plant survival and even continued growth.

The architecture of AR is not only determined by environmental signals but is also under strong genetic control. In the dark, ARs grow upwards presumably in a quest to obtain more oxygen [37]. Turbid floodwaters greatly restrict light penetration into the water, thereby minimizing the influence of light on directional root growth, and the light intensity decreases further with depth. The architecture of the AR system is comprehensively affected by altered gas diffusion, light intensity, and gravity. A number of genes have been characterized to be involved in crown root development and these genes participate in different molecular networks [44,45,46]. Crown roots and adventitious roots of rice have not been systematically distinguished because they both originate from the arial node. We thereby assumed that the regulation of crown roots and adventitious roots largely share overlapping gene networks, and we focus the remaining discussion on aboveground ARs.

It is believed that the aboveground AR root system is influenced by the following three parameters: (*i*) Position (number) of the aboveground node. AR primordia are present at the node and, therefore, more nodes result in the emergence of numerous ARs during flooding. The stem nodes in the majority of rice cultivars are buried in the soil or grow near the soil surface, whereas deepwater rice grows more aboveground nodes. The overexpression of several genes significantly increased the number of aboveground nodes such as *WUSCHEL-related homeobox gene 11* (*WOX11*) [47], *Mao Hu Zi 4* (*MHZ4*) [48], *SNORKEL1* (*SK1*), and *SNORKEL2* (*SK2*) [4]. (*ii)* The number of root primordia at each node. The number of root primordia at each node differs between rice genotypes. In addition, the number of AR is controlled by root-related genes such as *CROWN ROOTLESS 1* (*CRL1*) [49], *CROWN ROOTLESS 5* (*CRL5*) [50], *WOX11* [47], *TRYPTOPHAN AMINOTRANSFERASE OF ARABIDOPSIS1* (*TAA1*) [51] etc. (*iii*) The response to ethylene. Ethylene is required for adventitious root emergence, and the rapid induction of functional ARs is beneficial for plants to survive at the early flooding stage.

## 5. Discussion, Conclusions and Future Perspectives

One individual root trait is likely insufficient to substantially improve crop adaptation to flooding [52]. Thus, we propose that two or three adaptive traits should be simultaneously introduced into the target crops in order to provide sufficient flood tolerance. In traditional rice breeding, QTLs or valuable haplotypes of candidate genes can be introgressed into a modern high-yielding cultivar through cross breeding. However, such breeding approaches are usually time-consuming, laborious, and natural variations are unavailable for some traits. By contrast, modern genetic engineering holds the potential to greatly speed up the process.

Aerenchyma and ROL barrier induction occur at the onset of waterlogging and can therefore be seen as a flood response helping soil roots to adapt to short-term flooding soon after flooding kicks in. However, as the flooding progresses, a gradual functional loss of soil roots results from oxygen deficiency and accumulation of phytotoxins in the flooded soil [23]. The establishment of a new root system replacing the soil roots to maintain water and nutrient uptake is therefore an essential adaptive mechanism enabling survival and growth during continuous and periodic submergence (Figure 2B). Even if several environmental signaling molecules have been identified [53], the intra-tissue molecular mechanism of the ROL barrier induction in plants is still poorly understood. Nevertheless, we anticipate that rice varieties, which are tolerant to both short-term and long-term flooding, will be developed in the future.

In stark contrast, the molecular mechanisms of aerenchyma development and AR growth have been comprehensively studied and are therefore much better understood. Development of both inducible aerenchyma and AR growth are triggered by ethylene and mediated via ROS accumulation, suggesting a shared overlapping gene network. Uncovering the functional genes regulating both traits will be crucial for breeding of flood-tolerant rice.

The gene network involved in AR development and growth has been systematically investigated [45,46], and the marker genes can be applied as major indicators for molecular breeding in agriculture. To date, the most extensively reported genes related to AR development are regulated by the phytohormone auxin and cytokinin [46]. To fully understand the role or potential application of these genes, it is commonly tested by overexpression in rice. Plants constitutively expressing these genes oftentimes cause phytohormone disorder, thus leading to plant growth defects. Constitutive overexpression of *OsYUC* in plants exhibits more ectopic ARs but at the expense of a dwarf phenotype [51]. Root growth is regulated genetically and by environmental factors. Roots with the potential to emerge from aboveground primordia are supposed to grow only when triggered by soil flooding, because roots lacking a cuticle are prone to desiccation in low humidity. Overexpression of *WOX11* in rice shows more nodes and emerged ARs even in emergent conditions, which is seemingly not appropriate for the plant [47]; a similar observation has been made in the *ABA4* rice mutant [48]. It is thus believed that driving these genes with hypoxia-inducible promoters may be preferable for the breeding of flood-tolerant rice. *Submergence 1A* (*Sub1A*) [54], *SNORKEL1* (*SK1*), and *SNORKEL2* (*SK2*) [4] are flooding tolerance-related genes detected in several wildtypes of rice. Their expressions are exclusively induced during soil flooding and the corresponding promoters can be cloned and further exploited. In addition, the expression of many other genes is also highly and specifically upregulated during soil flooding, such as *Pyruvate Decarboxylase* (*PDC*) [55] and *Alcohol Dehydrogenase* (*ADH*) [55], and their promoters are highly feasible as well [56].

The majority of the molecular elements characterizing adaptive root traits are positive regulators. Unfortunately, there are relatively few reports with respect to negative regulators and it is clear that biased research methods have resulted in ignoring the functions of these negative regulators. We therefore propose to pay more attention in the future to those negative regulatory factors related to the three traits. Owing to the development of cluttered regularly interspaced short palindromic repeat (CRISPR)/CRISPR-associated protein (Cas) system and the gradual liberalization of genome editing crops [57], knocking out these important negative factors is not only conducive to basic scientific research but could have a large potential in crop application. Scientists from China have modified six important agronomic traits of wild allotetraploid rice through genome editing, making it possible to de novo domesticate wildtype plants [58]. It has been revealed that the rice gene *Os8N3* (*OsSWEET11*) negatively regulates plant resistance to *Xanthomonas* and the knockout of *Os8N3* significantly enhanced plant tolerance to *Xanthomonas* [59]. CRISPR/Cas9-targeted mutagenesis of the *OsRR22* gene has also been shown to significantly improve salinity tolerance [60]. These cases clearly indicate that the technology is feasible and reliable when used to modify adaptive traits under biotic and abiotic stress. An alternative strategy is to induce the specific nucleotide substitution of C to T or G to A in the regulatory elements at the promoter region; thereby, the expression of these identified positive regulators can be manipulated. However, the gain-of-function results are relatively hard to achieve, even though the predictable nucleotide-specific mutations can be realized through base editing using CRISPR/Cas technology. Moreover, the efficiency of such approaches remains low [61].

In addition to the three key root traits covered in the present review, several other traits have been described and will likely be involved in future crop improvement, including root porosity [18] and internode aerenchyma formation [6]. Similar to root aerenchyma, the root porosity enhances the internal aeration and facilitates oxygen diffusion to the root tip where it sustains respiration. Aerenchyma also develops in the internode of rice, where constitutive aerenchyma is primarily formed in the older internodes and further induced at the upper internode indicative of a developmental manner. Based on the research on 18 wild Poaceae species, three important indicators were developed to reflect plant root adaptation to soil flooding, including cortex to stele ratio (CSR), xylem to stele ratio (XSR), and aerenchyma to cortex ratio (ACR) [62]. These three key root parameters can be used for breeding flood-tolerant crops in the future. Because it is time-consuming to breed flooding-tolerant rice varieties through traditional hybridity, accurate targeted gene or QTLs might significantly shorten the breeding time. The development of these adaptive traits aims at either enhancing O_2_ uptake or improving O_2_ use efficiency. In the present review, we propose a breeding strategy involving the introgression of two or even three different flooding adaptive traits into high-yielding modern rice cultivars. Such an approach would lead to the improvement of plant survival and therefore reduce grain loss during soil flooding.

## Data Availability

Not applicable.
